# Clinical Accuracy of Instrument-Read SARS-CoV-2 Antigen Rapid Diagnostic Tests (Ag-IRRDTs)

**DOI:** 10.1155/2022/9489067

**Published:** 2022-05-09

**Authors:** Ali Umit Keskin, Pinar Ciragil, Aynur Eren Topkaya

**Affiliations:** ^1^Department of Biomedical Engineering, Yeditepe University, Istanbul, Turkey; ^2^Department of Microbiology, Yeditepe University Kozyatagi Hospital, Istanbul, Turkey; ^3^Department of Microbiology, Yeditepe University Kosuyolu Hospital, Istanbul, Turkey

## Abstract

This systematic review (PROSPERO registration number: CRD42021282476) aims to collect and analyse current evidence on real-world performance based on clinical accuracy of instrument-read rapid antigen diagnostic tests (Ag-IRRDTs) for SARS-CoV-2 identification. We used PRISMA Checklist and searched databases (PubMed, Web of Science Core Collection and FIND) for publications evaluating the accuracy of SARS-CoV-2 Ag-IRRDTs as of 30 September 2021, and included 40 independent clinical studies resulting in 48 Ag-IRRDT datasets with 137,770 samples. Across all datasets, pooled Ag-IRRDT sensitivity was 67.1% (95% CI: 65.9%–68.3%) and specificity was 99.4% with a tight CI. Pooled sensitivity and specificity of SARS-CoV-2 Ag-IRRDTs did not demonstrate a significant superiority over SARS-CoV-2 rapid antigen tests which do not require a reader instrument, even in the case where surveillance and screening datasets were excluded from the analysis. Nevertheless, they provide connectivity advantages and remove operator interface (in results-reading) issues. The lower sensitivity of certain brands of Ag-IRRDTs can be overcome in high prevalence areas with high frequency of testing. New SARS-CoV-2 variants are major concern for current and future diagnostic performance of these tests.

## 1. Introduction

Since the World Health Organization declared COVID-19 a pandemic in March 2020, rapid and accurate testing for SARS-CoV-2 become essential for clinical management and effective isolation of COVID-19 patients. While qRT-PCR instruments detect viral nucleic acid in few hours, and are considered the gold standard for detecting COVID-19, they have high purchasing and running costs and require dedicated staff to operate.

Both Ag-RDTs and Ag-IRRDTs are rapid, low cost, portable, and simple to operate devices which can be used in point-of-care (POC) use as well as in hospitals, schools and sports communities. FIAs constitute a subset of Ag-IRRDTs. An Ag-IRRDT device provides user-independent test results. Since there is an electro-optical reader of the Ag-IRRDT, there is a possibility of connection to laboratory-information system in the hospitals which provide ease of documentation and archiving, but they are also amenable to point-of-care testing. Ag-IRRDT assays consist of lateral flow cartridges where the specimens are manually loaded. Results are read on a small portable electronic reader. These devices are considered easy to use without much training required. However, they are not suitable for batch testing, as (in most cases) only a single sample can be analyzed at a time and the device requires 3–20 min process period, in addition to about 5-min disinfection and drying procedure [1]. It should be noted that (although FIAs dominate the Ag-IRRDT world market at present), a definition of Ag-IRRDT does not exclusively imply operation of tests using fluorescence principle alone, since a reader instrument may sense the lines on the sensor cassette by visible reflectance principle. A Comparison of reported SARS-CoV-2 probes can be found elsewhere [2, 3]. However, currently only few of them are employed in commercially available instruments.

Earlier review articles on Ag-RTDs in the literature either do not include Ag-IRRDTs [4, 5] or have limited coverage [6–9]. Another study [10] presents an extended review of Ag-RTDs but mixed Ag-IRRDTs with CLEIAs.

Reports and guidelines of regulatory agencies [11–13] and healthcare authorities [14, 15] also take the performances of Ag-IRRDTs into consideration in varied depth scale.

This SR attempts to give a current overview of manufacturer independent studies for an objective assessment of Ag-IRRDTs, applying some specific inclusion criteria, as of 30, September, 2021. To the best of our knowledge, present study is a unique systematic review in the literature which has been concentrated specifically on Ag-IRRDTs. Such independent reviews can be helpful in differentiation studies of test devices eligible for reimbursement in worldwide healthcare systems.

## 2. Methods

### 2.1. Survey Methodology

**Figure 1:**
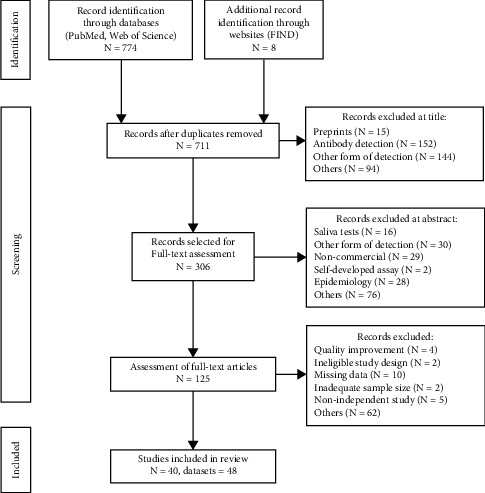
PRISMA flow-diagram showing systematic study processing (FIND: https://www.finddx.org/covid-19/).

The PRISMA flow-diagram [16] and standard guidelines for systematic reviews were followed as shown in Figure 1. Additionally, the systematic review was registered on PROSPERO (Registration number: CRD42021282476).

### 2.2. Search Strategy

Databases PubMed, Web of Science Core Collection, as well as the Foundation for Innovative New Diagnostics (FIND) website were searched using the terms of SARS-CoV-2, COVID-19, coronavirus, evaluation, accuracy, point of care testing, POC tests, fluorescence immunoassay, fluorescence, FIA and rapid antigen test. Two authors (A.E. and A.U.K) performed the Search Strategy. Disagreements were resolved by continued discussions until a unanimous decision was reached in a session with the participation of all authors, third author (P.C.) acting as a referee.

### 2.3. Inclusion Criteria

Only peer-reviewed publications and reports were included (preprints were not included in the analysis). No language restrictions were applied. If existed, publications with a tested sample population size of less than 30 were excluded.

Studies based on saliva samples were excluded in this study due to evidence regarding use of saliva as Ag-RDT specimen type has conflicting results [12].

While new brands of rapid antigen test devices for SARS-CoV-2 enter into the market, their performances were reported to be markedly lower than the manufacturers' specs [17–19]. Hence independent analyses are essential for accurate judgement of device performances. Assessment of independence from manufacturers was based on whether a study received financial support from a test manufacturer or any study author was affiliated with a test manufacturer. Here, only those independent (non-manufacturer sponsored) Ag-IRRDT-based studies were included. Reagent, device and other consumable materials donations were exempt from exclusion decision.

Only those studies which clearly report sample size, sensitivity and specificity of their measurements were included in this analysis with qRT-PCR as the reference standard.

This SR takes the assumption that qRT-PCR testing is the most appropriate measure of comparison for the diagnosis of COVID-19. While viral culture might provide better measurements, it suffers from other implementation issues. Nevertheless, some studies reporting their results in reference to viral culture were also included in the SR.

Descriptive analyses of all studies were performed to estimate pooled sensitivity and specificity in comparison to qRT-PCR testing.

### 2.4. Data Extraction and Analysis

Studies were screened, their characteristics were extracted independently by each reviewer. Each of the reviewers were acting blind during this process. Two reviewers (A.U.K and A.E, A.U.K and P.C., or A.E and P.C.) reviewed the titles and abstracts of all publications independently, then followed by a full-text review for those eligible, to select the articles for inclusion in this study. Any disagreements were resolved by the participation of third reviewer in joint discussions.

The last name of the first author of a study was used along with the country where testing took place, the manufacturer and model names of the Ag-IRRDT kits, total number of subjects, sample condition (fresh or un-fresh), sample types (NP, MT, OP, AN), compliance with manufacturer instructions for use (IFU), the number of positive qRT-PCR samples, reported sensitivity and specificities and ranges of Ct values of the reference standard. The results were tabulated using a reference number for each dataset. Pooled data results were also given.

Sensitivity and specificity for each test were presented with 95% confidence intervals (CIs). Data extraction was independently performed using 2-by-2 contingency tables of the number of true positives, false positives, false negatives and true negatives, and data according to viral load (high or low, according to Ct cut-offs defined within studies) were separately extracted.

The results were presented using the forest plots of sensitivity and specificity, in each case. Pooled sensitivities and specificities were computed according to test manufacturer.

### 2.5. Statistical Analysis and Data Synthesis

Raw data were extracted from the studies and performance estimates were recalculated. Forest plots indicating sensitivity and specificity and their CIs for each test, as well as for polled sensitivity and specificity and their CIs are plotted. Then, the heterogeneity between studies was visually evaluated. Accuracy parameters and their CIs were recalculated. In order to assess the uncertainty introduced by sample size, the 95% CIs were calculated using Wilson's method.

A group-analysis was performed for a test group if three or more datasets were available under its title, otherwise only a descriptive analysis was performed, and sensitivity-specificity ranges were reported.

Point estimates of accuracy parameters for SARS-CoV-2 detection were reported relative to their qRT-PCR results with 95% confidence intervals (CIs). The meta-analyses and relevant plots were constructed by using “metafor” package and a bivariate model package “mada” in *R* 4.0.1 software (*R* Foundation for Statistical Computing, Vienna, Austria) and *R*Studio (*R*Studio, Inc., Boston, MA, USA) (version 1.3).

Sample type assessment was accomplished using nasopharyngeal (NP) alone against combined oropharyngeal (OP), anterior nasal (AN) or mid-turbinate (MT) specimens, grouped as “others.”

### 2.6. Methodological Quality Assessment and Publication Bias

Assessment of the quality of the included studies were independently performed by two authors (A.E. and P.C) using the diagnostic test accuracy quality assessment tool of the Joanna Briggs Institute (https://jbi.global/critical-appraisal-tools). Discrepancies were resolved in a discussion session with the participation of all authors. Quality (risk of bias) grading were accomplished as follows: Total score ≤49; low-quality (high-risk of bias), total score 50–69: moderate-quality (moderate-risk of bias); total score ≥70%, high-quality (low risk of bias). Funnel plots were constructed to detect publication bias.

### 2.7. Sensitivity Analysis

Estimation of sensitivity and specificity analysis was planned by excluding surveillance and screening studies. The results of each sensitivity analysis were compared against overall results to assess the potential bias introduced by considering surveillance and screening studies.

### 2.8. Analytical Comparisons

This study design was confined to clinical diagnostic studies, therefore a comparison with analytical studies was beyond the scope of this SR.

### 2.9. Comparing Performances of SARS-CoV-2 Ag-RDTs against SARS-CoV-2 Ag-IRRDTs

We searched earlier papers that present an overview of commercial SARS-CoV-2 Ag-RDTs not requiring a reading instrument. We then compared the performance results of studies dealing with SARS-CoV-2 Ag-IRRDTs against earlier SRs which report performances of commercial SARS-CoV-2 Ag-RDTs not requiring a reading instrument.

### 2.10. Comparing Performances of SARS-CoV-2 Ag-IRRDTs against Combination of SARS-CoV-2 Ag-RDTs and Ag-IRRDTs

As another benchmarking, the overall sensitivity measure reported in other SRs which include both SARS-CoV-2 Ag-RDTs and Ag-IRRDTs was compared with the overall sensitivity measure reported in this SR (which includes only SARS-CoV-2 Ag-IRRDTs).

## 3. Results

### 3.1. Summary of Studies

This SR included 48 clinical accuracy datasets reported in 40 sources with a total number of 137,770 samples and 5,925 samples with confirmed SARS-CoV-2 by qRT-PCR.

### 3.2. Overall Performance of Ag-IRRDTs

Across all analysed samples, the pooled Ag-IRRDT sensitivity and specificity were 67.1% (95% CI 66.7% to 69.1%) and 99.4% (95% CI 99.4% to 99.4%), respectively.

**Figure 2:**
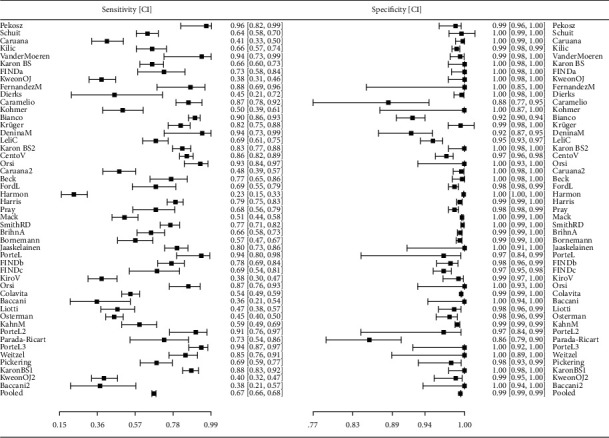
Forest Plots for all studies.Test for equality of sensitivities: *χ*^2^ = 875.98, df = 47, *p* < 0.01, Test for equality of specificities: *χ*^2^ = 1776.08, df = 47, *p* < 0.01. The list of studies shown here has the same order as in Table 1.

Table 1 displays all 48 datasets gathered on the Ag-IRRDT based studies that were eligible in this SR.  Figure 2 shows forest plots of these 48 tests included in this SR, as well as their pooled result (Accuracy estimates with 95% confidence interval were calculated using the Wilson score method).

Diagnostic odds ratio (for all 48 tests combined) is computed as DOR = 336.54 (95% CI = 308.09–367.61), positive likelihood ratio LR+ = 111.318 (95% CI = 103.63–119.58), and negative likelihood ratio LR− = 0.331 (95% CI = 0.319–0.343) along with the test for equality of sensitivities: *χ*2 = 875.98, df = 47, *p* < 0.01 test for equality of specificities: *χ*2 = 1776.08, df = 47, *p* < 0.01 indicated that overall heterogeneity of the tests was high.

### 3.3. Methodological Quality Assessment

The diagnostic test accuracy quality assessment tool of the Joanna Briggs Institute diagnostic accuracy checklist was used (with 480 entries with 48 resulting scores) to examine the quality of each study that has been included in this SR. The highest quality score of the included studies was 88.9/100 (12 studies). The lowest quality score was 55.6/100 (4 studies). Overall, there were no low-quality studies, 62.5% of high-quality, and 37.5% of moderate-quality studies.

### 3.4. Publication Bias

**Figure 3:**
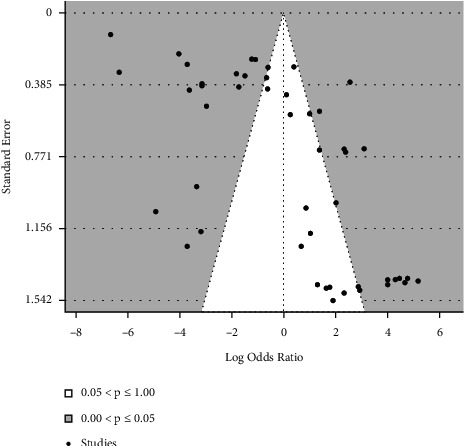
Funnel plot for all datasets. Effect size was taken as log odds ratio. Egger's test results were as follows: Random-Effects Model (*k* = 48), *τ*^2^ (estimated amount of total heterogeneity): 8.3860 (SE = 1.9011), *I*^2^ (total heterogeneity/total variability): 97.84%, *H*^2^ (total variability/sampling variability): 46.22, Test for Heterogeneity: Q (df = 47) = 2580.13, *p* < 0.0001.

For the publication bias assessment, a funnel plot is drawn including all datasets of this SR along with the results of Egger's tests are shown in Figure 3. In this plot, the effect size was taken as the logarithm of odds ratio.

Regression Test for funnel plot asymmetry (using weighted regression with multiplicative dispersion model and standard error as predictor) yields *t* = 5.7391, *p* < 0.0001 and limit estimate value of intercept is *b* = −5.3701 (CI: −6.4775, −4.2626). Inspection of the size of intercept shows that it differs significantly from zero, indicating funnel plot asymmetry, hence (possible) publication bias.

### 3.5. Prevalence of SARS-CoV-2

Prevalence rate (the number of qRT-PCR positive samples within the study population) varied between 0.4% and 78.7%. Pooled prevalence rate was 4.3%. However, it was noted that the prevalence of SARS-CoV-2 in most of these studies did not reflect the prevalence in the local populations, hence introducing a bias in the studies.

### 3.6. Symptomatic and Asymptomatic COVID-19 Population

Although most of the datasets reported in studies included in this SR were related to symptomatic COVID-19 cases, majority of samples were collected from asymptomatic individuals. This is because of the fact that three datasets alone [1, 40, 48] were surveillance studies including a total number of 107,514 Ag-IRRDT samples, comprising 78% of overall sample count and having 638 qRT-PCR verified positive cases in total.

### 3.7. Conformity with Manufacturers' Instructions for Use

It was noted that 22 studies reported conformity with the manufacturers' instructions for use of Ag-RRDTs out of 48 datasets (45.8%). Pooled sensitivity was 0.703 (95% CI: 0.686–0.720) and pooled specificity was 0.995 (95% CI: 0.994–0.995). Although surveillance and screening studies were present, these sub-group accuracy values are slightly higher than the overall accuracies of Ag-RRDTs. Diagnostic odds ratio and likelihood ratios of pooled tests were as follows: DOR = 439.44 (95% CI: 391.38–493.40), LR+ = 131.15 (95% CI = 120.60–142.62) and LR− = 0.30 (95% CI: 0.28–0.32).

Test for equality of sensitivities yields *χ*^2^ = 325.36, and for specificities *χ*^2^ = 925.33, both with df = 21 and *p* < 0.01 indicate the existence of heterogeneity in this sub-group of Ag-IRRDT studies which reported conformity to manufacturers' instructions. In this sub-group, correlation between sensitivities and false positive rates was weak (*ρ* = 0.183 with 95% CI: −0.259–0.561).

Pooled accuracies of non-conforming sub-group including 26 datasets were computed as follows: Sensitivity = 0.645 (95% CI: 0.629–0.661), specificity = 0.990 (95% CI: 0.989–0.992). The non-conforming sub-group accuracy values were lower than the overall accuracies of Ag-RRDTs. In this sub-group, test for equality of sensitivities yield *χ*^2^ = 526.4, and test for equality of specificities provide *χ*^2^ = 570.9, both with df = 26 and *p* < 0.01, indicating substantial heterogeneity.

**Figure 4:**
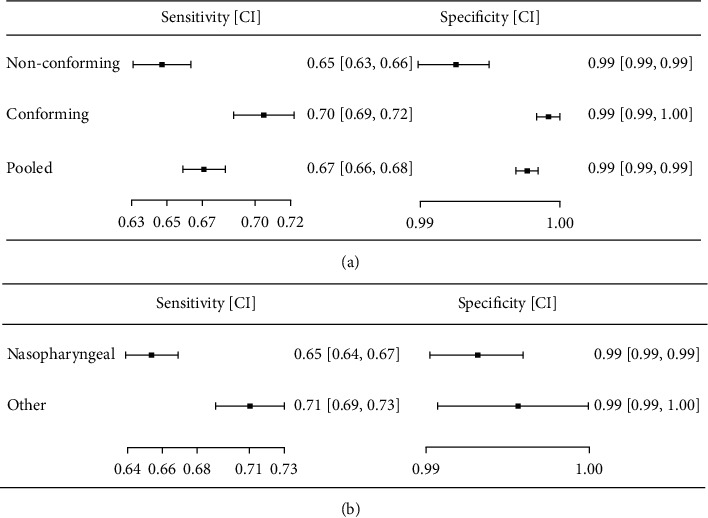
(a) Forest plots related to manufacturers' “instructions for use” conformity of two study subgroups of instrument-read rapid diagnostic antigen tests. Overall pooled result of the tests is also included for reference. (b) Forest diagrams relating sample types used in Ag-IRRDTs. 32 Nasopharyngeal tests against 16 other sample types (Anterior-Nasal, Mid-turbinate, Oropharyngeal) of tests.

Figure 4(a) displays the forest plots related to conformity to manufacturers' instructions for use of Ag-IRRDTs.

### 3.8. Analysis by Sample Type

Nasopharyngeal (NP) samples with oropharyngeal (OP), anterior nasal (AN) or mid-turbinate (MT) swab samples, or with their combinations were assessed to categorize tests by sample type. Note that saliva tests were excluded in this SR. The most common sample type evaluated was NP swabs (in 32 studies, 66.7%) followed by AN (in 7 studies, 14.6%). Hence, NP swab samples were separately analysed for their accuracy performance against other sample types.  Figure 4(b) displays forest plots related to sample types. NP swab samples achieved a pooled sensitivity of 0.651 (95% CI: 0.635–0.665). DOR = 300.9 (95% CI: 271.3–333.9) and test results for equality of sensitivities in pooled NP swabs (*χ*^2^ = 512.4, *p* < 0.01) demonstrate heterogeneity in sensitivity values for tests done using NP swabs.

### 3.9. Analysis by Sample Condition

Pooled sensitivity values of Ag-IRRDTs for un-fresh and fresh samples were 66.9% (95% CI: 64%–70%) and 67.2% (95% CI: 66%–68%), respectively.

### 3.10. Ag-IRRDT Sensitivity by Ct Value

This is used as a surrogate for viral load to estimate the limit of detection of antigen tests. A single Ct threshold value of Ct = 30 was selected and sensitivities of available datasets were investigated according to specified threshold, rather than using multiple Ct values. As expected, all Ag-IRRDTs showed higher sensitivity values in samples with high viral loads, and sensitivity dropped beyond Ct >30 (Table 2).

### 3.11. Sensitivity Analysis

When analysis was restricted to studies that exclude three surveillance and screening reports, overall pooled sensitivity increased from 67.1% to 69.3% (95% CI: 68% to 70.5%) and overall pooled specificity decreased from 99.4% to 98.7% (95% CI: 98.5% to 98.8%).

### 3.12. Meta-Regression

A meta-regression was not performed due to substantial heterogeneity in reporting subgroups.

### 3.13. Manufacturer Based Accuracies

Overall pooled sensitivity of five different Ag-IRRDT brands with the available database of more than three studies, altogether comprising 40 clinical accuracy datasets with 135,624 samples was 68.3%. Pooled specificity of the same sub-group was 99.4%. In this sub-group, correlation coefficient of sensitivities and false positive rates is *ρ* = 0.843.

**Figure 5:**
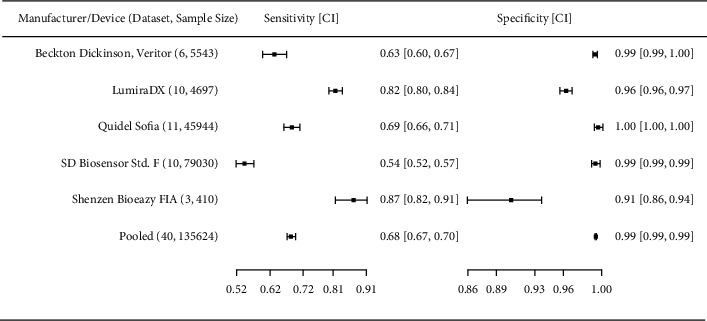
Forest diagrams for 5 different brands of Ag-IRRDTs. With the pooled diagnostic odds ratio DOR = 353.097, 95% CI: [322.423–386.688] and tests for equality of sensitivities and for specificities all show that heterogeneity of datasets for major Ag-IRRDT manufacturers is high. Although Shenzen Bioeasy FIA demonstrated the highest sensitivity value over 87%, the number of studies using this test and their pooled sample size (3, 410) were low. Note that its 95% CIs have the widest ranges for both sensitivity and specificity parameters. LumiraDX demonstrated a sensitivity of 81.8% with larger number of studies and samples (10 and 4,697, respectively) with relatively narrow ranges of CIs. Surveillance studies were included in Quidel Sofia and SD Biosensor Standard F Quidel Sofia showed highest specificity value. (Values are displayed in two-digit accuracy).

Figure 5 displays forest plots of these Ag-IRRDT brands. Eyeball test on forest plots and pooled diagnostic odds ratio DOR = 353.097, (95% CI: 322.423–386.688), positive likelihood ratio of LR+ = 112.514 (95% CI: 104.723–120.884), negative likelihood ratio of LR− = 0.319 (95% CI: 0.306–0.332), as well as test for equality of sensitivities calculated as *ρ*^2^ = 278.83, *p* < 0.01, and test for equality of specificities calculated as *χ*^2^ = 867.72, *p* < 0.01 show that heterogeneity in datasets for five major Ag-IRRDT manufacturers is high.

This SR highlights the top performance of the LumiraDX including 10 studies with pooled sensitivity of 81.8%, a sample size of 4,697 and with relatively narrow ranges of CIs for both sensitivity and specificity. Although Shenzen Bioeasy FIA demonstrated the highest sensitivity value of 87.2%, the number of studies and sample size (3, 410) were low. Note that its 95% CIs have the widest ranges for both sensitivity and specificity. SD Biosensor Standard F group had the highest number of test samples (79,030). Removing surveillance studies from SD Biosensor Standard F group did not change the pooled sensitivity value (54.4%) and reduced the pooled specificity from 99.4% to 98.5%. On the other hand, removing surveillance studies from Quidel Sofia demonstrated an increase of pooled sensitivity value from 68.7% to 74.6% (and a decrease in pooled specificity value from 99.7% to 98.5%) for 11,500 samples, placing Quidel Sofia among good performers.

### 3.14. Results of Comparing Performances of SARS-CoV-2 Ag-RDTs against SARS-CoV-2 Ag-IRRDTs

Hayer et al. [5] present an overview of commercial SARS-CoV-2 Ag-RDTs not requiring a reading instrument with 19 studies investigating five different Ag-RDTs presented detailed population characteristics and Ct values. Only three commercial Ag-RDTs have been assessed in multiple studies, and of these, only two brands had adequate levels of performance; their sensitivity estimates were around 80%. These two Ag-RDTs with the available database of more than eight studies, reported a specificity of 97% in the majority of the trials.

On the other hand, present SR includes more than 12 times the number of samples, 2.5 times the number of different all peer-reviewed studies and more than twice the number different brands with respect to earlier study [5] which did not include mass-surveillance reports, as shown in Table 3. Top performers of our SR include one brand with 10 datasets with pooled sensitivity of 81.8%, a sample size of 4,697 and with relatively narrow ranges of CIs for both sensitivity and specificity. Another good performer of our SR presents the highest sensitivity value of 87.2%, with 3 datasets and 410 samples.

### 3.15. Results of Comparing Performances of SARS-CoV-2 Ag-IRRDTs against Combination of SARS-CoV-2 Ag-RDTs and SARS-CoV-2 Ag-IRRDTs

Pooled sensitivity measure reported in another SR [10] was compared with the pooled sensitivity reported in this SR, when the datasets from preprints (about 37% of their dataset count) were excluded. In this case, the new sensitivity value was reported as 0.672 (95% CI: 0.629–0.713) which came close to the value of overall pooled sensitivity reported in our present study. However, it should be noted here that, this new sensitivity value is for the overall combination of Ag-RDTs and Ag-IRRDTs.

## 4. Discussion

Lower sensitivities of Ag-IRRDT tests are due to false-negative results in some patients. Therefore, any negative result for a symptomatic patient should be confirmed by qRT-PCR test. This reduces the clinical utility of rapid antigen tests in low prevalence areas. Nevertheless, Ag-IRRDT tests can be useful in areas where molecular testing is not available or overloaded.

It should be noted here that it is currently unclear how test positivity (by any test) translates into clinical infectiousness and person-to-person spread [52].

Ag-IRRDT tests may vary in analytical sensitivity. This is one reason for differing clinical sensitivities of these tests. It was shown that [23] the relationship between Ct and viral load was poor for samples with Ct values >33. The large variation of clinical sensitivities between different brands of Ag-IRRDTs could also be due to individual study design, operator competencies and quality of the Ag-IRRDT itself. The lower sensitivity demonstrated by certain brands of Ag-IRRDTs can be overcome in high prevalence areas with high frequency of testing that may partly relieve some concerns around sensitivity [7, 57].

In reference to qRT-PCR validation, ideal Ag-IRRDT sensitivity as a function of Ct value would be a flat curve. However, this is not the case in practice, and sensitivity decreases as Ct value increases. The rate of decrease in sensitivity happens to be at a faster pace beyond a certain Ct level. Thus, the likelihood of false-negative antigen test results becomes higher at lower viral loads. While some studies detected no difference in the mean Ct values between symptomatic patients and asymptomatic patients [41], others reported that symptomatic patients displayed lower Ct values than asymptomatic COVID-19 patients, and a Ct value of 30 is the threshold for SARS-CoV-2 infectivity [22, 38]. Moreover, it was shown [37] that different sensitivity versus Ct value patterns prevail in symptomatic and asymptomatic patient groups.

It should be noted here that all measurement conditions cannot be expected to be the same for every study. For example, measurement temperature may also affect Ag-IRRDT sensitivity and specificity results [58], but only few reports include their measuring temperature ranges. Similarly, a lack of evidence to guide optimal nasal swab testing can increase the risk of false-negative test results [59]. Whether SARS-CoV-2 antigen-detection using a rapid test with self-collected nasal swab or professional-collected nasopharyngeal swab makes a difference can be another issue [60]. Cross-reactivity from other viral samples (like dengue, syphilis, hepatitis B and rheumatoid factor) are usually not considered by most researchers. Currently, most disturbing parameter is the existence of new SARS-CoV-2 variants [61] that may adversely affect rapid antigen test performance [62]. It should be pointed out here that the sensitivity of any COVID-19 tests to new SARS-CoV-2 variants were not considered in the studies included in this review.

As the research on specific problems [63–65] related to COVID-19 is exponentially growing, use of reliable, cost effective and fast means of diagnosing the disease become very valuable. In order to meet this need, numerous non-molecular tests such as SARS-CoV-2 Ag-RDTs and SARS-CoV-2 Ag-IRRDTs have been introduced by different manufacturers in the worldwide market. The SR presented in this paper have shown that (contrary to expectations), overall pooled sensitivity and specificity of SARS-CoV-2 Ag-IRRDTs did not demonstrate a significant superiority over SARS-CoV-2 Ag-RDTs which do not require a reader instrument, even in the case where surveillance and screening datasets were excluded from the analysis. Nevertheless, they provide connectivity advantages and reduce operator interface (reading) issues.

One possible limitation of the present SR design is the assumption (as in the previously published SRs) that qRT-PCR testing is the standard measure of reference. Viral culture might provide a better measure of comparison; however, it suffers considerable implementation problems in practice. In addition, the present SR did not assess the influences of age, gender, symptom duration and sample collector (a swab sample obtained by a trained professional or a self-collected swab) on the accuracy of Ag-IRRDTs.

## 5. Conclusions

Most manufacturers of Ag-IRRDTs can produce high specificity tests, but their sensitivities are low and there are significant differences in their sensitivity (15%–99%). The lower sensitivity of certain brands of Ag-IRRDTs can be overcome in high prevalence areas with high frequency of testing. Conformity to the manufacturers' instructions for use in testing procedure improves the accuracy of these tests. New SARS-CoV-2 variants are major concern and they should be evaluated in the future studies.

## Figures and Tables

**Table 1:** Clinical sensitivity and specificity data for instrument-read rapid antigen tests (Ag-IRRDTs) against COVID-19.

Ref No	First Author Name	Lab. Country	Mfr	Sample type	Fresh sample	IFUC	D	Sensitivity	Sensitivity CI	Specificity	Specificity CI
[18]	Pekosz	USA	1	NP	Y	N	251	0,96400	0,823–0,994	0,98700	0,961–0,995
[19]	Schuit	Holland	1	NP	Y	N	2678	0,63900	0,576–0,698	0,99600	0,992–0,998
[20]	Caruana	Swiss	1	NP	Y	N	532	0,41200	0,326–0,504	0,99800	0,987–1,000
[21]	Kilic	USA	1	AN	Y	Y	1384	0,66400	0,574–0,743	0,98800	0,981–0,993
[22]	VanderMoeren	Holland	1	MT-OP	N	N	351	0,94100	0,730–0,990	0,99400	0,978–0,998
[23]	Karon BS	USA	1	NP	N	N	347	0,66500	0,596–0,727	1,00000	0,975–1,000
[24]	FINDa	Swiss	2	NP	Y	Y	232	0,73200	0,581–0,843	1,00000	0,980–1,000
[25]	KweonOJ	Korea	2	NP	N	N	322	0,38300	0,313–0,459	1,00000	0,976–1,000
[26]	FernandezM	Spain	3	NP	Y	Y	46	0,87500	0,690–0,957	1,00000	0,851–1,000
[27]	Dierks	Germany	3	NP	Y	Y	444	0,45500	0,213–0,720	0,99500	0,983–0,999
[28]	Caramelio	Italy	3	AN	Y	Y	149	0,86600	0,784–0,920	0,88500	0,770–0,946
[29]	Kohmer	Germany	3	NP	Y	N	100	0,50000	0,389–0,611	1,00000	0,871–1,000
[30]	Bianco	Italy	3	AN	Y	N	907	0,90300	0,864–0,931	0,92100	0,897–0,940
[31]	Krüger	Germany	3	NMT	Y	Y	761	0,82200	0,752–0,875	0,99300	0,983–0,997
[32]	DeninaM	Italy	3	NP	N	N	191	0,94100	0,730–0,990	0,91900	0,869–0,951
[33]	LeliC	Italy	3	NP	N	N	792	0,68700	0,613–0,752	0,95200	0,932–0,966
[23]	Karon BS2	USA	3	NP	N	N	347	0,83200	0,774–0,878	1,00000	0,975–1,000
[34]	CentoV	Italy	3	NP	Y	Y	960	0,85600	0,815–0,889	0,97200	0,956–0,983
[35]	Orsi	Italy	4	NP	Y	Y	110	0,93300	0,841–0,974	1,00000	0,929–1,000
[20]	Caruana2	Swiss	5	NP	Y	N	532	0,48300	0,393–0,573	0,99500	0,983–0,999
[36]	Beck	USA	6	AN	Y	Y	346	0,77000	0,651–0,858	0,99600	0,980–0,999
[37]	FordL	USA	6	NMT	Y	Y	1051	0,68500	0,553–0,793	0,98500	0,975–0,991
[1]	Harmon	USA	6	AN	Y	Y	23462	0,22900	0,152–0,330	0,99870	0,998–0,999
[38]	Harris	USA	6	AN	Y	Y	2436	0,79300	0,746–0,834	0,99300	0,988–0,996
[39]	Pray	USA	6	AN	Y	Y	1098	0,68420	0,553–0,786	0,98460	0,975–0,991
[40]	Mack	USA	6	NP	N	N	10982	0,51150	0,438–0,585	0,99630	0,995–0,997
[41]	SmithRD	USA	6	NP	Y	Y	2887	0,76600	0,708–0,816	0,99740	0,995–0,999
[42]	BrihnA	USA	6	NP	Y	Y	2039	0,65800	0,578–0,728	0,99380	0,989–0,996
[43]	Bornemann	Germany	6	NP	Y	Y	1391	0,57100	0,469–0,668	0,99300	0,987–0,996
[44]	Jaaskelainen	Finland	6	NP	N	N	188	0,80400	0,733–0,860	1,00000	0,912–1,000
[45]	PorteL	Chile	6	NP-OP	N	N	64	0,93800	0,799–0,983	0,96900	0,843–0,994
[46]	FINDb	Brazil	7	NP	Y	Y	453	0,77500	0,692–0,841	0,97900	0,957–0,990
[46]	FINDc	Germany	7	NP	Y	Y	676	0,69200	0,536–0,814	0,96900	0,952–0,980
[47]	KiroV	India	7	NP	Y	Y	354	0,38200	0,305–0,466	0,99000	0,967–0,997
[35]	Orsi	Italy	7	NP	Y	Y	110	0,86700	0,758–0,931	1,00000	0,929–1,000
[48]	Colavita	Italy	7	NP	Y	Y	73070	0,54331	0,493–0,593	0,99455	0,994–0,995
[49]	Baccani	Italy	7	NP	N	N	93	0,35700	0,207–0,542	1,00000	0,944–1,000
[50]	Liotti	Italy	7	NP	N	N	359	0,47000	0,378–0,566	0,98400	0,960–0,994
[51]	Osterman	Germany	7	NP-OP	N	N	741	0,45400	0,405–0,504	0,97800	0,957–0,989
[52]	KahnM	Germany	7	NP-OP	N	N	3110	0,59375	0,494–0,687	0,98970	0,985–0,993
[45]	PorteL2	Chile	7	NP	N	N	64	0,90600	0,758–0,968	0,96900	0,843–0,994
[53]	Parada-ricart	Spain	8	NP	Y	Y	172	0,73100	0,539–0,863	0,85600	0,790–0,904
[54]	PorteL3	Chile	8	NP-OP	N	N	127	0,93900	0,865–0,974	1,00000	0,921–1,000
[55]	Weitzel	Chile	8	NP-OP	N	N	111	0,85000	0,756–0,912	1,00000	0,890–1,000
[56]	Pickering	UK	9	AN-OP	N	N	200	0,69000	0,594–0,772	0,98000	0,930–0,994
[30]	KaronBS1	USA	10	NP	N	N	347	0,88300	0,831–0,921	1,00000	0,975–1,000
[25]	KweonOJ2	Korea	11	NP	N	N	322	0,39500	0,324–0,471	0,98700	0,954–0,996
[49]	Baccani2	Italy	11	NP	N	N	81	0,37500	0,212–0,573	1,00000	0,937–1,000

Total number of samples is 137,770, pooled sensitivity = 0.671 (95% CI: 0.659–0.683), pooled specificity = 0.994 (95%CI:0.994–0.994). *D* = Number of samples, Mfr = Manufacturer code: (1) Beckton, Dickinson and Co., Veritor USA; (2) Boditech Med., iChroma, COVID-19 Ag, Chuncheon-si, Gang-won-do, Korea; (3) Lumira Dx Limited, London, UK; (4) NanoEntek FREND, Guro-gu, Seoul, Korea; (5) Precision Biosensor Exdia, Daejeon, Korea; (6) Sofia FIA Quidel Corporation, San Diego, CA, USA; (7) Standard F, SD Biosensor, Suwon, Korea; (8)Shenzen Bioeasy Biotechnology, Shenzhen, Guangdong Province PRC; (9) Surescreen FIA, Derby DE1 3QB, UK; (10) FIAflex SARS-CoV-2 Antigen FIA ACON Biotech (Hangzhou) Co., Ltd. PRC; (11) Boditech Med., AFIAS COVID-19 Ag, Chuncheon-si, Gang-won-do, Korea.

**Table 2:** Sensitivities extracted from 13 different Ag-IRRDT performance evaluation studies related to 15 cases for reference qRT-PCR values of Ct <30 (waived between 29 and 33) which yields an increase in pooled sensitivity value of the group from 0.73 to 0.85.

First author [ref]	Manufacturer	Description	Ct <	Sensitivity
V. Moeren [22]	BD veritor	<7 days	30	0,983
V. Moeren [22]	BD veritor	>7 days	30	0,854
Kweon [25]	Boditech iChroma		30	0,891
Baccani2 [49]	Boditech AFIAS		30	0,643
Kweon2 [25]	Boditech AFIAS		30	0,740
Krüger [31]	LumiraDX		30	0,902
Cento [34]	LumiraDX		29	0,908
Harris [38]	Quidel sofia	Symptomatic, Ag+	30	0,968
Harris [38]	Quidel sofia	Symptomatic, Ag−	30	0,630
Harris [38]	Quidel sofia	Asymptomatic, Ag−	30	0,077
Jaaskelainen [44]	Quidel sofia		30	0,943
FINDb [46]	SD biosensor F		33	0,809
FINDc [46]	SD biosensor F		33	0,750
Baccani [49]	SD biosensor F		30	0,714
Kahn [52]	SD biosensor F		30	0,642
Pooled Ct < 30				0,854

**Table 3:** Comparison of two systematic review studies. Our study presenting only instrument-read rapid antigen tests is compared and contrasted with another review^4^ that included only rapid antigen tests which does not employ any reader equipment. Positive sample counts are those tests that were confirmed by qRT-PCR.

Property	Ag-RDTs	Ag-IRRDTs
Reference	Hayer et al. [5]	This study
Number of datasets	19	48
Number of samples	11,109	137,770
Number of manufacturers	5	11
As of date:	20.11.2020	30.09.2021
Positive sample count	2,509	5,925
Sensitivity min. %	28.9	22.9
Sensitivity min. 95% CI	16.4–44.3	15.2–33.0
Sensitivity max. %	98.3	96.4
Sensitivity max. 95% CI	91.1–99.7	82.3–99.4
Specificity min.%	92.4	85.6
Specificity min. 95% CI	87.4–95.9	79.0–90.4
Specificity max.%	100.0	100.0
Specificity max. 95% CI	99.7–100.0	98.0–100.0
Max. pooled sensitivity, %	82.4	87.2
Max. pooled sens., 95% CI	87.4–95.9	81.2–91.3
Pooled sensitivity, %	74.7	67.1
Pooled sens., 95% CI	67.3–80.9	65.9–68.3
Min.-max. Prevalence, %	1.9–100	0.4–78.7
Higgins' *I*^2^%	93	10.75

## Data Availability

Data available from corresponding author upon reasonable request.

## Abbreviations

Ab: Antibody
Ag: Antigen
Ag-RDT: Antigen rapid diagnostic test
Ag-IRRDT: Instrument-read antigen rapid diagnostic test
AN: Anterior Nasal
*b*: Limit estimate value of intercept (An indicator of funnel plot asymmetry)
CI: Confidence interval (taken as 95% of indicated parameter value)
CLEIA: Chemiluminescent Immunoassay
COVID-19: Coronavirus disease 2019 caused by SARS-CoV-2
Ct: Cycle threshold
DOR: Diagnostic odds ratio
df: Degree of freedom
FIA: Fluorescence immunoassay
IFU: Instructions for use
JBI: Joanna Briggs Institute (University of Adelaide)
*χ* ^2^: Chi-squared value
LR: Likelihood ratio
MT: Mid-turbinate
NP: Nasopharyngeal
OP: Oropharyngeal
POC: Point of Care
qRT-PCR: Quantitative Reverse-Transcriptase Polymer Chain Reaction
*ρ*: Correlation coefficient
ROC: Receiver Operating Characteristic
SARS-CoV-2: Severe Acute Respiratory Syndrome Coronavirus-2
SE: Standard Error.

## References

[1] Harmon K., de St Maurice A. M., Brady A. C. (2021). Surveillance testing for SARS-COV-2 infection in an asymptomatic athlete population: a prospective cohort study with 123,362 tests and 23,463 paired RT-PCR/antigen samples. *BMJ Open Sport & Exercise Medicine*.

[2] Karakuş E., Erdemir E., Demirbilek N., Liv L. (2021). Colorimetric and electrochemical detection of SARS-CoV-2 spike antigen with a gold nanoparticle-based biosensor. *Analytica Chimica Acta*.

[3] Aydın E. B., Aydın M., Sezgintürk M. K. (2021). Highly selective and sensitive sandwich immunosensor platform modified with MUA-capped GNPs for detection of spike Receptor Binding Domain protein: a precious marker of COVID 19 infection. *Sensors and Actuators B: Chemical*.

[4] Aguilar-Shea A. L., Vera-García M., Güerri-Fernández R. (2021). Rapid antigen tests for the detection of SARS-CoV-2: a narrative review. *Atención Primaria*.

[5] Hayer J., Kasapic D., Zemmrich C. (2021 Jul). Real-world clinical performance of commercial SARS-CoV-2 rapid antigen tests in suspected COVID-19: a systematic meta-analysis of available data as of November 20, 2020. *International Journal of Infectious Diseases*.

[6] Graham M., Ballard S. A., Pasricha S. (2021). Use of Emerging Testing Technologies and Approaches for SARS-CoV-2: Review of Literature and Global Experience in an Australian Context. *Pathology*.

[7] Mistry D. A., Wang J. Y., Moeser M. E., Starkey T., Lee L. Y. W. (2021). A systematic review of the sensitivity and specificity of lateral flow devices in the detection of SARS-CoV-2. *BMC Infectious Diseases*.

[8] Dinnes J., Deeks J. J., Berhane S. (2021). Rapid, point-of-care antigen and molecular-based tests for diagnosis of SARS-CoV-2 infection. *Cochrane Database of Systematic Reviews*.

[9] Khandker S. S., Nik Hashim N. H. H., Deris Z. Z., Shueb R. H., Islam M. A. (2021). Diagnostic accuracy of rapid antigen test kits for detecting SARS-CoV-2: a systematic review and meta-analysis of 17,171 suspected COVID-19 patients. *Journal of Clinical Medicine*.

[10] Brümmer L. E., Katzenschlager S., Gaeddert M. (2021). Accuracy of novel antigen rapid diagnostics for SARS-CoV-2: a living systematic review and meta-analysis. *PLoS Medicine*.

[11] Taher J., Randell E. W., Arnoldo S. (2021). Canadian Society of Clinical Chemists (CSCC) consensus guidance for testing, selection and quality management of SARS-CoV-2 point-of-care tests. *Clinical Biochemistry*.

[12] Bohn M. K., Lippi G., Horvath A. R. (2021). IFCC interim guidelines on rapid point-of-care antigen testing for SARS-CoV-2 detection in asymptomatic and symptomatic individuals. *Clinical Chemistry and Laboratory Medicine*.

[13] European Commission (2021). Current performance of COVID-19 test methods and devices and proposed performance criteria. https://ec.europa.eu/docsroom/documents/40805?locale=es.

[14] World Health Organization (2021). Antigen-detection in the diagnosis of SARS-CoV-2 infection using rapid immunoassays. https://www.who.int/publications/i/item/antigen-detection-in-the-diagnosis-of-sars-cov-2infection-using-rapid-immunoassays.

[15] https://www.fda.gov/medical-devices/coronavirus-covid-19-and-medical-devices/removal-lists-tests-should-no-longer-be-used-andor-distributed-covid-19-faqs-testing-sars-cov-2.

[16] Moher D., Liberati A., Tetzlaff J., Altman D. G., The PRISMA Group (2009). Preferred reporting items for systematic reviews and meta-analyses: the PRISMA Statement. *PLoS Medicine*.

[17] Kipritci Z., Keskin A. Ü., Çıragil P., Topkaya A. E. (2021). Evaluation of a visually-read rapid antigen test kit (SGA V-chek) for detection of SARS-CoV-2 virus. *Mikrobiyoloji Bulteni*.

[18] Pekosz A., Cooper C., Parvu V. (2020). Antigen-based testing but not real-time PCR correlates with SARS-CoV-2 virus culture. *Clinical Infectious Diseases*.

[19] Schuit E., Veldhuijzen I. K., Venekamp R. P. (2021). Diagnostic accuracy of rapid antigen tests in asymptomatic and presymptomatic close contacts of individuals with confirmed SARS-CoV-2 infection: cross sectional study. *BMJ*.

[20] Caruana G., Croxatto A., Kampouri E. (2021). Implementing SARS-CoV-2 Rapid antigen testing in the Emergency ward of a Swiss university hospital: the INCREASE study. *Microorganisms*.

[21] Kilic A., Hiestand B., Palavecino E. (2021). Evaluation of performance of the BD Veritor SARS-CoV-2 chromatographic immunoassay test in patients with symptoms of COVID-19. *Journal of Clinical Microbiology*.

[22] Van der Moeren N., Zwart V. F., Lodder E. B. (2021). Evaluation of the test accuracy of a SARS-CoV-2 rapid antigen test in symptomatic community dwelling individuals in The Netherlands. *PLoS One*.

[23] Karon B. S., Donato L. J., Bridgeman A. R. (2021). Analytical sensitivity and specificity of four point of care rapid antigen diagnostic tests for SARS-CoV-2 using real-time quantitative PCR, quantitative droplet digital PCR, and a mass spectrometric antigen assay as comparator methods. *Clinical Chemistry*.

[24] FINDa Foundation for Innovative New Diagnostics (2021). *FIND Evaluation of Boditech Medical, Inc. iChroma COVID-19 Ag Test. External Report Version 10*.

[25] Kweon O. J., Lim Y. K., Kim H. R. (2021). Evaluation of rapid SARS-CoV-2 antigen tests, AFIAS COVID-19 Ag and ichroma COVID-19 Ag, with serial nasopharyngeal specimens from COVID-19 patients. *PLoS One*.

[26] Fernández M. D., Estévez A. S., Alfonsín F. L., Arevalo G. B. (2021). Usefulness of the lumiradx™ sars-cov-2 antigen test in nursing home. *Enfermedades Infecciosas y Microbiología Clínica*.

[27] Dierks S., Bader O., Schwanbeck J. (2021). Diagnosing SARS-CoV-2 with antigen testing, transcription-mediated amplification and real-time PCR. *Journal of Clinical Medicine*.

[28] Caramello V., Boccuzzi A., Basile V. (2021). Are antigenic tests useful for detecting SARS-CoV-2 infections in patients accessing to emergency departments? Results from a North-West Italy hospital. *Journal of Infection*.

[29] Kohmer N., Toptan T., Pallas C. (2021). The comparative clinical per formance of four SARS-CoV-2 rapid antigen tests and their correlation to infectivity in vitro. *Journal of Clinical Medicine*.

[30] Bianco G., Boattini M., Barbui A. M. (2021). Evaluation of an antigen-based test for hospital point-of-care diagnosis of SARS-CoV-2 infection. *Journal of Clinical Virology*.

[31] Krüger L. J., Klein J. A. F., Tobian F. (2021). Evaluation of accuracy, exclusivity, limit-of-detection and ease-of-use of LumiraDx™: an antigen-detecting point-of-care device for SARS-CoV-2. *Infection*.

[32] Denina M., Giannone V., Curtoni A. (2021). Can we trust in Sars-CoV-2 rapid antigen testing? Preliminary results from a paediatric cohort in the emergency department. *Irish Journal of Medical Science*.

[33] Leli C., Di Matteo L., Gotta F. (2021). Performance of a SARS-CoV-2 antigen rapid immunoassay in patients admitted to the emergency department. *International Journal of Infectious Diseases*.

[34] Cento V., Renica S., Matarazzo E. (2021). Frontline screening for SARS-CoV-2 infection at emergency department admission by third generation rapid antigen test: can we spare RT-qPCR?. *Viruses*.

[35] Orsi A., Pennati B. M., Bruzzone B. (2021). On-field evaluation of a ultra-rapid fluorescence immunoassay as a frontline test for SARS-CoV-2 diagnostic. *Journal of Virological Methods*.

[36] Beck E. T., Paar W., Fojut L., Serwe J., Jahnke R. R. (2021). Comparison of the Quidel Sofia SARS FIA test to the hologic aptima SARS-CoV-2 TMA test for diagnosis of COVID-19 in symptomatic outpatients. *Journal of Clinical Microbiology*.

[37] Ford L., Lee C., Pray I. W. (2021). Epidemiologic characteristics associated with SARS-CoV-2 antigen-based test results, rRT-PCR cycle threshold values, subgenomic RNA, and viral culture results from university testing. *Clinical Infectious Diseases*.

[38] Harris D. T., Badowski M., Jernigan B. (2021). SARS-CoV-2 rapid antigen testing of symptomatic and asymptomatic individuals on the university of Arizona campus. *Biomedicines*.

[39] Pray I. W., Ford L., Cole D. (2021). Performance of an antigen-based test for asymptomatic and symptomatic SARS-CoV-2 testing at two university campuses - Wisconsin, september-october 2020. *MMWR-Morbidity and Mortality Weekly Report*.

[40] Mack C. D., Osterholm M., Wasserman E. B. (2021). Optimizing SARS-CoV-2 surveillance in the United States: insights from the national football league occupational Health program. *Annals of Internal Medicine*.

[41] Smith R. D., Johnson J. K., Clay C. (2021). Clinical evaluation of Sofia Rapid Antigen Assay for detection of severe acute respiratory syndrome coronavirus 2 (SARS-CoV-2) among emergency department to hospital admissions. *Infection Control & Hospital Epidemiology*.

[42] Brihn A., Chang J., OYong K. (2021). Diagnostic performance of an antigen test with RT-PCR for the detection of SARS-CoV-2 in a hospital setting - los angeles county, California, june-august 2020. *MMWR-Morbidity and Mortality Weekly Report*.

[43] Bornemann L., Kaup O., Kleideiter J., Panning M., Ruprecht B., Wehmeier M. (2021). Real-life evaluation of the Sofia SARS-CoV-2 antigen assay in a large tertiary care hospital. *Journal of Clinical Virology*.

[44] Jääskeläinen A. E., Ahava M. J., Jokela P. (2021). Evaluation of three rapid lateral flow antigen detection tests for the diagnosis of SARS-CoV-2 infection. *Journal of Clinical Virology*.

[45] Porte L., Legarraga P., Iruretagoyena M. (2021). Evaluation of two fluorescence immunoassays for the rapid detection of SARS-CoV-2 antigen—new tool to detect infective COVID-19 patients. *PeerJ*.

[46] Findb (2020). *FIND Evaluation of SD Biosensor, Inc. STANDARD™ F COVID-19 Ag FIA. External Report Version 21*.

[47] Kiro V. V., Gupta A., Singh P. (2021). Evaluation of COVID-19 antigen fluorescence immunoassay test for rapid detection of SARS-CoV-2. *Journal of Global Infectious Diseases*.

[48] Colavita F., Vairo F., Meschi S. (2021). COVID-19 rapid antigen test as screening strategy at points of entry: experience in lazio region, Central Italy, August-October 2020. *Biomolecules*.

[49] Baccani I., Morecchiato F., Chilleri C. (2021). Evaluation of three immunoassays for the rapid detection of SARS-CoV-2 antigens. *Diagnostic Microbiology and Infectious Disease*.

[50] Liotti F. M., Menchinelli G., Lalle E. (2021). Performance of a novel diagnostic assay for rapid SARS-CoV-2 antigen detection in nasopharynx samples. *Clinical Microbiology and Infections*.

[51] Osterman A., Baldauf H. M., Eletreby M. (2021). Evaluation of two rapid antigen tests to detect SARS-CoV-2 in a hospital setting. *Medical Microbiology and Immunology*.

[52] Kahn M., Schuierer L., Bartenschlager C. (2021). Performance of antigen testing for diagnosis of COVID-19: a direct comparison of a lateral flow device to nucleic acid amplification-based tests. *BMC Infectious Diseases*.

[53] Parada-Ricart E., Gomez-Bertomeu F., Pico´-Plana E., Olona-Cabases M. (2020). Usefulness of the antigen for diagnosing SARS-CoV-2 infection in patients with and without symptoms. *Enfermedades Infecciosas Y Microbiologia Clinica*.

[54] Porte L., Legarraga P., Vollrath V. (2020). Evaluation of a novel antigen-based rapid detection test for the diagnosis of SARS-CoV-2 in respiratory samples. *International Journal of Infectious Diseases*.

[55] Weitzel T., Legarraga P., Iruretagoyena M. (2021). Comparative evaluation of four rapid SARS-CoV-2 antigen detection tests using universal transport medium. *Travel Medicine and Infectious Disease*.

[56] Pickering S., Batra R., Merrick B. (2021). Comparative performance of SARS-CoV-2 lateral flow antigen tests and association with detection of infectious virus in clinical specimens: a single-centre laboratory evaluation study. *The Lancet Microbe*.

[57] Larremore D. B., Wilder B., Lester E. (2021). Test sensitivity is secondary to frequency and turnaround time for COVID-19 screening. *Science Advances*.

[58] Haage V., Ferreira de Oliveira-Filho E., Moreira-Soto A. (2021). Impaired performance of SARS-CoV-2 antigen-detecting rapid diagnostic tests at elevated and low temperatures. *Journal of Clinical Virology*.

[59] Callesen R. E., Kiel C. M., Hovgaard L. H. (2021). Optimal insertion depth for nasal mid-turbinate and nasopharyngeal swabs. *Diagnostics*.

[60] Lindner A. K., Nikolai O., Kausch F. (2021). Head-to-head comparison of SARS-CoV-2 antigen-detecting rapid test with self-collected nasal swab versus professional-collected nasopharyngeal swab. *European Respiratory Journal*.

[61] Keskin A. U., Bolukcu S., Ciragil P., Topkaya A. E. (2021). SARS-CoV-2 specific antibody responses after third CoronaVac or BNT162b2 vaccine following two-dose CoronaVac vaccine regimen. *Journal of Medical Virology*.

[62] Bourassa L., Perchetti G. A., Phung Q. (2021). A SARS-CoV-2 nucleocapsid variant that affects antigen test performance. *Journal of Clinical Virology*.

[63] Zandi M., Farahani A., Zakeri A. (2021). Clinical symptoms and types of samples are critical factors for the molecular diagnosis of symptomatic COVID-19 patients: a systematic literature review. *International Journal of Microbiology*.

[64] Malekifar P., Pakzad R., Shahbahrami R. (2021). Viral coinfection among COVID-19 patient groups: an update systematic review and meta-analysis. *BioMed Research International*.

[65] Soltani S., Zakeri A., Zandi M. (2021). The role of bacterial and fungal human respiratory microbiota in COVID-19 patients. *BioMed Research International*.

